# Serum MicroRNA-155 as a Potential Biomarker to Track Disease in Breast Cancer

**DOI:** 10.1371/journal.pone.0047003

**Published:** 2012-10-10

**Authors:** Yu Sun, Minjie Wang, Guigao Lin, Shipeng Sun, Xuexiang Li, Jun Qi, Jinming Li

**Affiliations:** 1 National Center for Clinical Laboratories, Beijing Hospital of the Ministry of Health, Beijing, People’s Republic of China; 2 Clinical Laboratories, Cancer Hospital, Chinese Academy of Medical Sciences, Beijing, People’s Republic of China; 3 Graduate School, Peking Union Medical College, Chinese Academy of Medical Sciences, Beijing, People’s Republic of China; 4 Clinical Laboratories, Guang’anmen Hospital, China Academy of Chinese Medical Sciences, Beijing, People’s Republic of China; Okayama University, Japan

## Abstract

**Background:**

One major impediment to improving the management of breast cancer is the current lack of tumor marker with sufficient sensitivity and specificity. A growing body of evidence implicates the diagnostic potential of circulating miRNAs in cancer detection. MiR-155 plays an important role in the pathogenesis of breast cancer. However, the level of circulating miR-155 and its clinical relevance are not well established. The objective of the current study was to learn more about serum miR-155 in patients with breast cancer.

**Methodology/Principal Findings:**

Using quantitative reverse transcription polymerase chain reaction (RT-qPCR), we demonstrated that serum miR-155 had significant increased levels in breast cancer patients (n = 103) compared with healthy subjects (n = 55) (*p*<0.001), which had a mean fold change of 2.94. Receiver operating characteristic (ROC) analysis revealed that miR-155 had considerable diagnostic accuracy, yielding an ROC-AUC (the areas under the ROC curve) of 0.801 (sensitivity 65.0%, specificity 81.8%). In addition, sera from a subset of breast cancer patients (n = 29) were collected after surgery and after four cycles of chemotherapy to evaluate the effects of clinical treatment on serum levels of candidate miRNAs. Surprisingly, a decreased level of serum miR-155 was found; whereas the concentrations of carbohydrate antigen 15-3 (CA15-3), carcinoembryonic antigen (CEA) and tissue polypeptide specific antigen (TPS) did not show this trend. Our results revealed that 79% patients showed response or stable disease after therapy had declined levels of serum miR-155.

**Conclusions/Significance:**

Our results suggest that serum miR-155 is a potential biomarker to discriminate breast cancer patients from healthy subjects. For the first time, we demonstrated a declined trend of miR-155 after surgery and chemotherapy, which raises the possibility to use it as an indicator for treatment response.

## Introduction

Breast cancer remains one of the top threats to the health of women. To date, carbohydrate antigen 15-3 (CA 15-3) is the most widely applied serum marker. However, the lack of sensitivity precluded its clinical use in early stage disease. For example, the levels of CA 15-3 are increased in ∼10% of patients with stage I disease and 20% with stage II disease [1]. Other serum markers such as carcinoembryonic antigen (CEA) and tissue polypeptide specific antigen (TPS) are even less sensitive than CA 15-3 [2], [3]. At present, CA 15-3 and CEA are mainly utilized to monitor therapy in metastatic breast cancer in combination with imaging, history and physical examination [3]–[5]. However, it should be taken into account that spurious rises of these markers may occur after the start of treatment without any clinical correlation [3]–[6].

Therefore, truly innovative approaches are highly desirable to move beyond the modest benefits achieved to date. Cell-free microRNAs (miRNAs) represent one of the novel strategies for cancer screening [7]–[10]. Using a xoengraft mouse model, it was demonstrated that there were cellular miRNAs released into the circulation [11]. Comparable expression of miRNAs in the circulation and tumor tissues was also implicated in the literature [12]–[15], suggesting the development of tumor directly contributes to the deregulation of circulating miRNA. Distinct biological properties, including remarkable stability, accessibility for rapid and accurate quantification, and a direct link with disease states [11], [16]–[17], make them ideally suited to serve as minimally invasive biomarkers to track disease. Recently some miRNA signatures have been described in breast cancer, including miR-155. Mir-155 is a robust oncogenic miRNA. It was reported that miR-155 downregulates *SOCS1* in breast cancer, in turn leading to persistent activation of STAT3 signaling [18]. The activation of inflammatory cascades thus indicates the communicative role of miR-155 between inflammation and cancer [18]. In breast cancer tissue, the overexpression of miR-155 was observed [19]. The oncogenic role and aberrant expression in tumor tissues raise the possibility that these miRNA may be deregulated in the circulation.

We notice that available data on the level of circulating miR-155 in breast cancer are inconsistently described. Zhu W et al. [20] firstly reported that the expression of serum miR-155 was comparable between cancer patients and healthy women; and tumors with positive progesterone receptor (PR) status had higher serum miR-155 expression than those were negative. A later study reported that increased level of serum miR-155 in subjects with invasive ductal carcinoma compared with health control; intriguingly, the author observed that higher level of miR-155 had a negative correlation with PR status [15]. Roth et al. [21] also found elevated expression of serum miR-155; however, serum samples were postoperatively collected, where the concentrations of miRNAs may decrease corresponding to the removal of tumor mass [22]. By contrast, Heneghan HM et al. [23] did not find significant difference between cancer patients and controls with regard to the level of miR-155 in whole blood. Furthermore, previous studies provided limited information about its diagnostic accuracy. Therefore, a full understanding of their expression and clinical significance is important before this knowledge is ultimately harnessed for therapeutic benefit.

In the first section of the current study, we screened the level of serum miR-155 in breast cancer patients, and examined its diagnostic accuracy. In the second section, we explored the influence of tumor resection and adjuvant chemotherapy on the expression of candidate miRNA, and presented discussions with an emphasis on its role in monitoring treatment response.

## Materials and Methods

### Ethics Statement

The study has been approved by the Ethics Committee of the National Center for Clinical Laboratories, and adhered to the tenets of the Declaration of Helsinki. In addition, written informed consent was obtained from each participant prior to sample collection.

### Study Design and Sample Collection

In the present study, we first screened the level of serum miR-155 in cases and controls; then we evaluated changes in levels of these miRNAs after curative resection and chemotherapy. Pre-operative sera from patients with histologically diagnosed breast cancer (n = 103) were drawn at Cancer Hospital, Chinese Academy of Medical Sciences from Oct 2010 to Sep 2011. Patient characteristics including age, TNM stage, T classification, nodal status, hormone receptors, HER2 overexpression, and subtype were retrospectively collected (Table 1). Patients with severe infection, active clinical comorbidities, or a history of any other malignancy were excluded. For 29 patients who underwent treatment, a second sample was obtained at the time of 1 month after tumor resection, and a third sample was collected at the periodical evaluation 3 months after the commencement of chemotherapy (Table S1). Applied adjuvant chemotherapies were epirubicin/cyclophosphamide, epirubicin/taxane, cyclophosphamide/pirarubicin, or fluorouracil/epirubicin/cyclophosphamide with and without taxane. In these patients, response to therapy was assessed by radiologists according to the World Organization (WHO) guidelines [24]. Additionally, sera from a set of 55 healthy females were collected from outpatients at Beijing Hospital. All participants were ethnic Chinese. None of healthy controls had previously diagnosed with any malignancies. The median age of these healthy subjects was 51 (range from 36 to 78). There is no significant difference of age between breast cancer and normal controls (*p* = 0.6999, Mann-Whitney U test). Blood sample from each participant was collected in tube with polymer gel and clot activator (BD Vacurainer® SST™ Tubes, Reference No. 367985). After clotting at room temperature for 30 min to 2 h, specimens were centrifuged at 1,300 g for 15 min. The supernatant was then centrifuged at 10,000 g for 10 min at 4°C to completely remove cellular contaminants. Sera were aliquoted into microcentrifuge tubes and stored at −80°C until use. Samples were aliquot and stored at −80°C before use.

**Table 1:** Patients information.

Characteristics	Breast cancer (n = 103)
**Age**	
Mean	51
Median (range)	53 (21–79)
**TNM stage**	
I	29
II	36
III	30
IV	8
**T classification**	
T_is_	3
T_1_	51
T_2_	42
T_3_	7
**Nodal status**	
Negative	43
Positive	60
**ER**	
Negative	34
Positive	63
Undetermined	6
**PR**	
Negative	27
Positive	64
Undetermined	12
**HER2**	
Negative	82
Positive	21
**Subtype**	
Luminal	63
HER2	8
Triple negative	20
Undetermined	12

Abbreviations: T_is_ = carcinoma in situ.

### RNA Extraction

RNA was isolated from 0.3 mL of serum by using the *mir*Vana PARIS Kit (Ambion) following the manufacturer’s instructions. Twenty pmol synthetic *C. elegans* miRNA (cel-miR-39) (Qiagen) was introduced after the addition of denaturing solution to teach sample to surveillance technical variations in RNA extraction, as has been described before [11]. Finally, RNA was recovered in 50 µL of RNase-free water. The RNA concentration was quantified by NanoDrop ND-1000 (Nanodrop, USA).

### Reverse Transcription (RT) and Quantitative Real-time PCR (qPCR)

RT and qPCR kits made specifically for accurate miRNA analysis (Applied Biosystems) were used to evaluate the expression of the miR-155 from serum samples. RT reactions were performed using the TaqMan microRNA Reverse Transcription Kit (Applied Biosystems, USA) in a final volume of 15 µL (incubated for 30 min at 16°C, 30 min at 42°C, 5 min at 85°C, and then maintained at 4°C). For real-time PCR, 3 µL diluted RT products were mixed with 10 µL of Taqman PCR master mixture (No UNG), 1 µL TaqMan MicroRNA Assay and 6 µL Nucleasefree water in a final volume of 20 µL. All reactions were preformed in triplicate on a 7500 Real-time system (Applied Biosystems) with the following conditions: 95°C for 10 min, followed by 40 cycles at 95°C for 15 s, and 60°C for 1 min.

Relative expression of miRNA was normalized to cel-mir-39, and was calculated using the 2^−△△CT^ method. ΔCT was calculated by subtracting the CT values of cel-miR-39 from the CT values of the target miRNAs. ΔΔCT was then determined by subtracting average ΔCT of the control from ΔCT of cases. The fold changes of candidate miRNA expression were calculated by the equation 2^−△△CT^ [25].

The term PCR efficiency is widely used in the context of standard curve made of a dilution series. In our study, standard curves were generated to determine whether PCR efficiency. The equation E = 10 ^(−1/slope)^ −1 was used to calculate PCR efficiency. As determined, the detection of miR-155 had a standard curve with a correlation coefficient of 0.999, a slope of - 3.356 and a PCR efficiency of and 98.6%; while the standard curve of cel-miR-39 had a correlation coefficient of 0.999 and a slope of −3.341 and the PCR efficiency of cel-miR-39 was 99.2%. The PCR efficiency is comparable between miR-155 and cel-miR-39.

To estimate the lower limit of detection, a series of 15 serial dilutions were produced by adding serially-2-fold-diluted synthetic cel-miR-39 into fifteen 400 µL serum samples (began with 2 pmol/L). The lower limit of detection of our study was 0.24 amol.

An estimate of inter-assay variation was obtained by analyzing eight replicates from three samples within a single assay. Inter-assay variation was obtained from the measurement of three samples performed on eight independent PCR runs. Because it is not appropriate to calculate variation based on Ct values generated from different runs [1], Ct values were converted into relative concentrations by the equation 2^−△△CT^. The inter-assay and intra-assay variation for the present study was less than 10.6% and 8.7%, respectively.

### Serum Assays for TPS, CEA, and CA15-3

We further compared the sensitivity and specificity of serum TPS, CEA, CA15-3, and miR-155. The levels of TPS, CEA, and CA15-3 were measured using commercially available kits (the Elecsys CEA and CA15-3 Immunoassay from Roche Diagnostics, Germany; TPS ELISA kit from IDL Biotech AB, Sweden); According to ROC curve analyses, the cut-off values are 66 U/L for TPS, 4.38 ng/mL for CEA, and 22 U/mL for CA15-3 (Figure S1). In our study, an increase or a decrease ≥25% in the serum marker levels was regarded as a significant alteration.

### Statistical Analysis

All statistical analyses were performed using SPSS 16.0 software (SPSS) and GraphPad Prism 5.0, GraphPad). Comparisons of serum miRNA levels were performed by applying Mann-Whitney U tests, Kruskal-Wallis tests, or Wilcoxon signed rank test when appropriate. Multiple hypothesis testing was adjusted by using Bonferroni correction. The Spearman rank order correlation test was used to examine associations between the levels of candidate miRNAs and clinical variables. Multivariate logistic regression model was developed, and odds ratio was used to evaluate risk factors. To assess the diagnostic accuracy, we performed receiver operating characteristic (ROC) curve analysis. The Area under the ROC curve (AUC) was then estimated. *P* values less than 0.05 were considered to be statistically significant.

## Results

### Analysis of Serum miR-155 in Subjects

**Figure 1:**
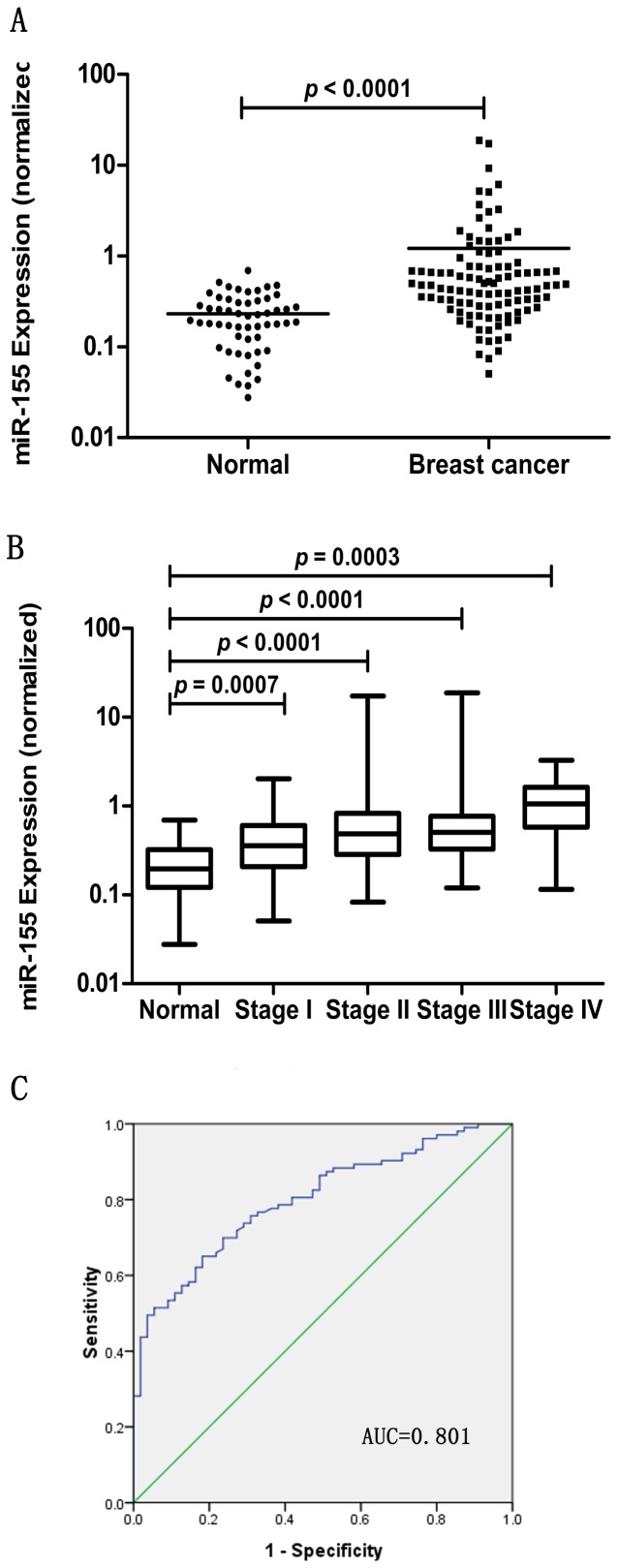
Serum miR-155 levels in normal controls (n = 55) and breast cancer patients (n = 103). The relative expression level of miR-155 was normalized to spiked-in cel-mir-39. The line represents the median value. Statistically significant difference was determined using Mann-Whitney tests. The result revealed a higher level of miR-155 in breast cancer patients (*p*<0.001) (A). Box plot of serum miR-155 expression levels across stages. The boxes indicate the 25th and 75th percentiles and the bold lines represent the median values. The relative expression levels of target miRNA were normalized to spiked-in cel-mir-39. Statistically significant differences were determined using Mann-Whitney tests or Kruskal-Wallis tests (B). Receiver operating characteristics (ROC) curve analysis for the diagnostic value of miR-155. The AUC (the areas under the ROC curve) was 0.801 (95% CI: 0.734 to 0.868) (C).

We first determined the expression of miR-155 in 158 serum samples (103 from patients with breast cancer and 55 from normal donors). Due to the lack of established internal control for miRNA RT-qPCR analysis in breast cancer, we used spiked-in cel-mir-39 for normalization. Consequently, the relative abundances of miR-155 were significantly upregulated in sera of cases compared with those of healthy controls (*p*<0.001, Mann Whitney test) (Figure 1A). Based on TNM staging, we stratified patients to examine the associations between serum levels of candidate miRNA and stages of disease. Of the 103 cases with breast cancer, miR-155 did not show significant difference across the staging (*p* = 0.0745 for miR-155, Kruskal-Wallis test). However, the levels of each individual tumor stage were markedly different from that of healthy controls (Figure 1B). In previous study, the levels of miR-155 have been highly correlated to hormone receptors positivity in sera from breast cancer patients [15], however, no significant difference was found in our study (*p* = 0.4519 for ER, and *p* = 0.1925 for ER, Mann Whitney test). Moreover, we failed to correlate the expression levels of miR-155 with other clinical parameters including tumor size (*p* = 0.0665, Kruskal-Wallis test), nodal status (*p* = 0.1421, Mann Whitney test), HER2 overexpression (*p* = 0.1238, Mann Whitney test) and molecular subtype (*p* = 0.3335, Kruskal-Wallis test).

### Diagnostic Value of Circulating miRNAs

ROC curve analysis showed that miR-155 was useful marker for discriminating cases from healthy controls, with an AUC (the areas under the ROC curve) of 0.801 (95% confidence interval (CI): 0.734 to 0.868, *p*<0.0001) (Figure 1C). At the cut-off value of 1.911, the optimal sensitivity and specificity were 65.0% and 81.8%, respectively. The odds ratio according to cut-off value was 8.956 (95% CI: 4.002–20.043).

### Monitoring of miR-155 during the Course of Treatment

**Figure 2:**
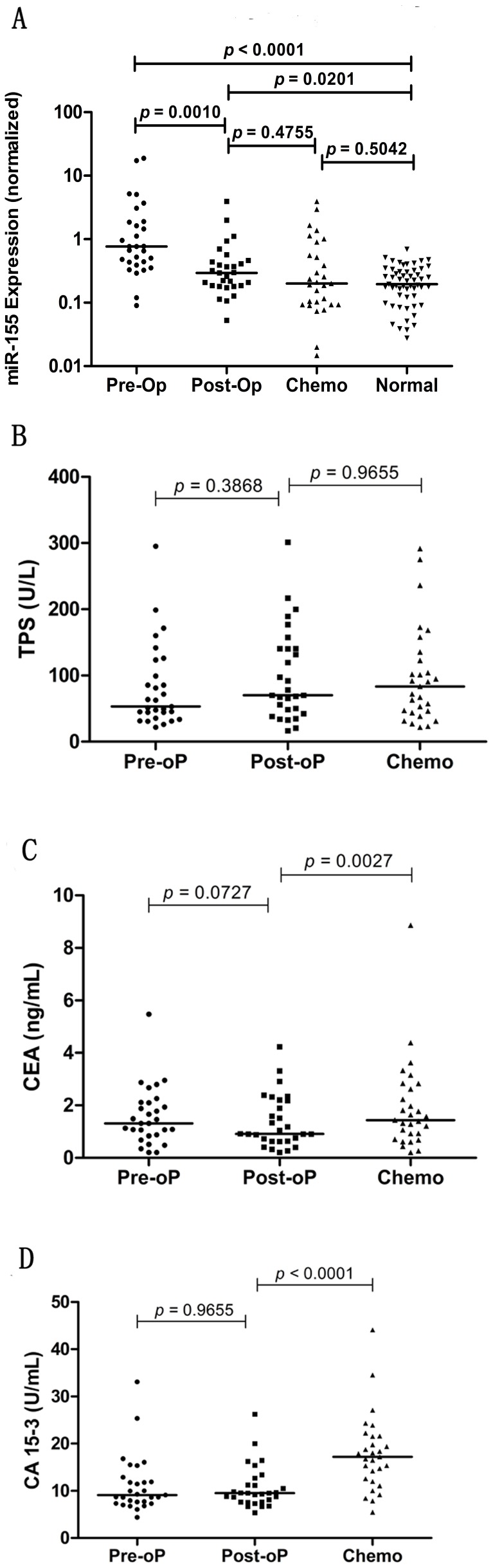
Changes in serum miR-155 and CA 15-3, CEA, TPS levels before (Pre-oP) and after surgery (Post-oP), and after chemotherapy (Chemo). Sera (n = 29) were collected from patients who underwent curative resection and adjuvant chemotherapy. The levels of miR-155 were significantly reduced after surgery (*p* = 0.0002), reaching levels comparable with healthy subjects (*p* = 0.5042) (A). The levels of TPS remained unchanged (*p*>0.05) (B). The levels of CA 15-3 and CEA underwent a significant elevation after chemotherapy (*p* = 0.0027 for CEA, *p*<0.0001 for CA 15-3) (C–D).

**Figure 3:**
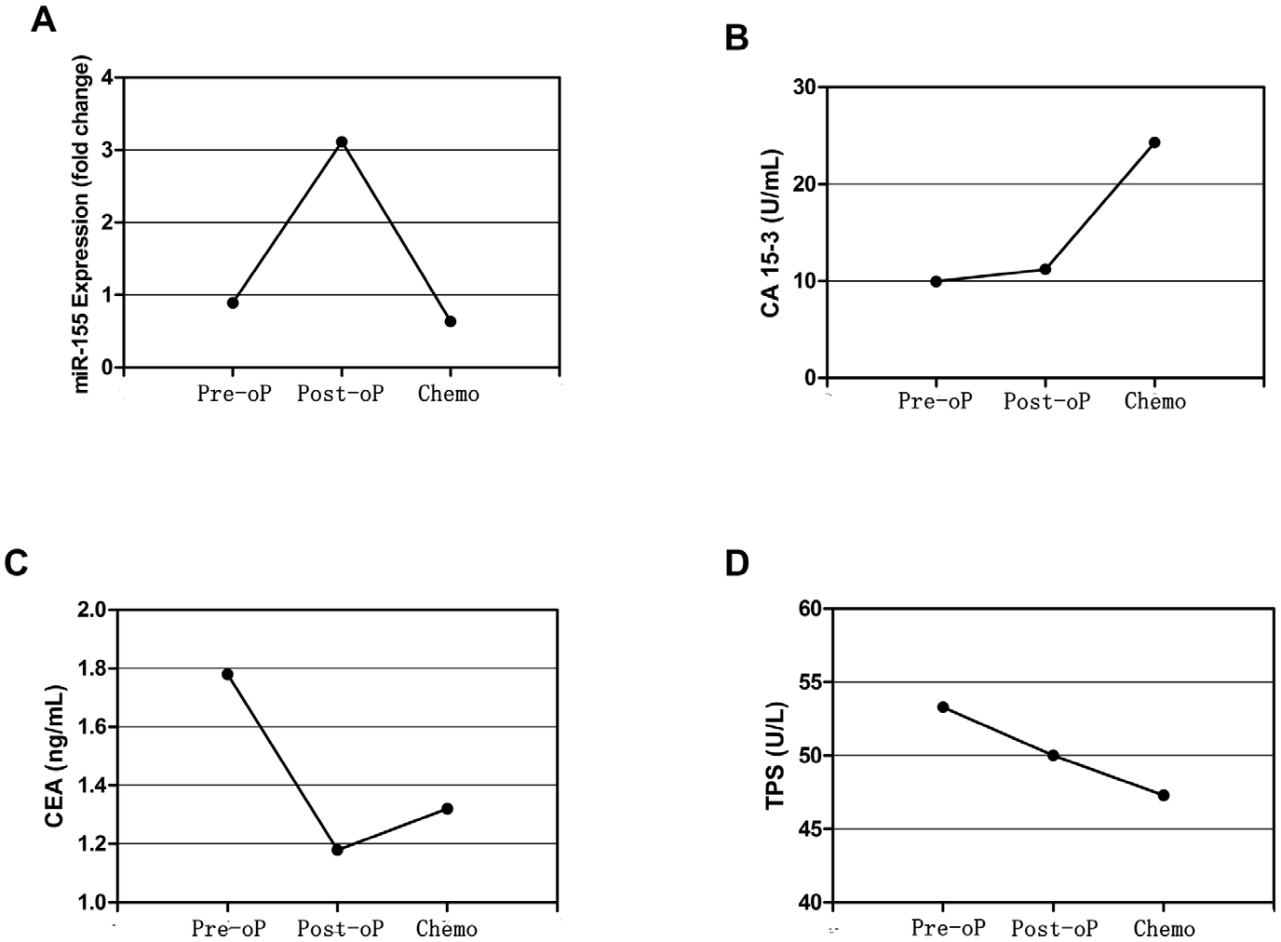
Changes in serum levels of miR-155, CA 15-3, CEA, and TPS in the patient who experienced relapse before (Pre-oP) and after surgery (Post-oP), and after chemotherapy (Chemo). The level of miR-155 underwent a 250% increase after surgery, and decreased to the preoperative level after chemotherapy (A). In light of a change in the serum marker levels ≥25% was regarded as a significant alteration, the level of CA 15-3 did not change after surgery but significantly elevated after chemotherapy (B); the level of CEA decreased after surgery (C); the level of TPS did not change (D).

Twenty-nine patients with nonmetastatic disease were screened for changes in concentrations of serum miR-155, TPS, CEA and CA 15-3 after surgery and adjuvant chemotherapy. The results showed that the levels of miR-155 underwent sharp decreases after treatment (*p* = 0.0016, Wilcoxon signed rank test), reaching levels comparable with healthy subjects (*p* = 0.5042, Mann Whitney test). The decreases have become evident after surgery (*p* = 0.0002, Wilcoxon signed rank test) (Figure 2A). We observed that 90% (n = 26) of all patients showed decreased levels of miR-155 after surgery, with a 73.1% median decrease (range between 10^th^ and 90^th^ percentiles, 10.7–95.5%); whereas three patients actually showed an increase (106%, 158% and 250%, respectively). The reason why the discharges of tumor lead to increased levels of miRNAs in these subjects has not been elucidated. However, a representative patient who developed lung metastasis after surgery may give us some clues. The level of miR-155 in this patient underwent a sharp increase (250%) after the end of therapy (Figure 3A); suggest increased expression of serum miR-155 may originate from surgically induced tissue injury or microscopic residues of the tumor. In addition, we did not find changes in the levels of miR-155 restricted to particular therapy schedules.

All subjects received four cycles of chemotherapy during the course of the study; thereafter blinded assessment of tumor response was performed by radiologists. All subjects experienced stable disease or remission of the tumor. In 79% (n = 23) of these subjects, the levels of miR-155 declined compared with preoperative miR-155 levels, with an 84.7% median decrease (range between 10^th^ and 90^th^ percentiles, 32.5–97.1%). While six patients showed different degrees of rises after the end of therapy (median 438%; range between 10^th^ and 90^th^ percentiles, 6–899%). In the patient who developed lung metastasis after surgery, chemotherapy resulted in the downregulation of serum miR-155 (Figure 3A).

### Comparison with TPS, CEA and CA 15-3

CA 15-3, CEA and TPS assays are routinely used for tracking diseases or monitoring treatment response. We explored the correlation between serum miR-155 levels and TPS, CEA, and CA15-3. A weak correlation between miR-155 and CA15-3 was found (r = 0.2291, *p* = 0.0206, Spearman correlation). According to their respective cut-off values, the optimal sensitivity and specificity were for 4.9% and 99.1% for CA 15-3, 7.8% and 98.2% for CEA, and 48.5% and 56.4% for TPS; whereas the optimal sensitivity and specificity of serum miR-155 was 65.0% and 81.8%. The difference in sensitivity between miR-155 and assays (CA15-3 or CEA or TPS) were statistically significant (*p*<0.001 for miR-155 versus CA 15-3, CEA or TPS, McNemar test).

For the first postoperative follow-up visit, the levels of CA 15-3, CEA and TPS did not change after tumor resection (*p* = 0.9655 for CA 15-3, *p* = 0.0727 for CEA, *p* = 0.3868 for TPS, Wilcoxon signed rank test); whereas after four cycles of chemotherapy, serum levels of CA 15-3 and CEA instead underwent an elevation (*p*<0.0001 for CA 15-3, *p* = 0.0727 for CEA, Wilcoxon signed rank test) (Figure 2B–D), compared with the declined trend of serum miR-155. There are 72.4% (21/29), 37.9% (11/29), and 55.2% (16/29) subjects showed increased expression of CA 15-3, CEA and TPS, respectively; only 20.7% (6/29) of all subjects showed increased expression of miR-155. These results suggest that these commonly applied assays are not sensitive to changes in tumor mass in this cohort of breast cancer. In the subject experienced increased expression of miR-155 after surgery, treatment resulted in different trends in the concentrations of CA 15-3, CEA and TPS (Figure 3B–D).

## Discussion

Currently, the identification of cancer-specific miRNA profiles in the circulation is an emerging field of particular interest. For the present study, we screened the level of circulating miR-155 in breast cancer by using RT-qPCR. The results showed that miR-155 was markedly increased in sera from breast cancer patients compared with normal controls; it had a mean fold change of 2.94. It is not surprising that the levels of circulating miR-155 were deregulated in breast cancer. A number of studies have built a critical role of miR-155 in breast carcinogenesis (Reviewed in 26, 27). Correspondingly, miR-155 has been observed to be overexpressed in breast cancer tissue [19], [28]. It is likely that the overexpressed miR-155 was released by tumor mass, as demonstrated in a xenograft mouse model [11]. It is of note that miR-155 is highly expressed in blood cells [29]. Since certain amount of miRNAs were derived from circulating blood cells, a question raises that if breast carcinogenesis has an indirect effect on blood cells, which leads to aberrantly released miR-155 in the circulation. Due to the poor-characterized mechanisms involving the origin of circulating miRNAs, it is hard to answer this question to date. It is possible that both proposals make sense. Despite performing a different normalization strategy, our results were comparable with those of Wang F et al. [15] and Roth C et al [21], who reported elevated levels of serum miR-155. The reason why Heneghan HM et al. [23] did not find significant difference in whole blood, as we proposed, is attributable to the choice of sample type; for their results may predominantly present miRNA profiles of blood cells. Due to a lack of diagnostic value of circulating miR-155 in former studies, we further performed ROC curve analysis. The result showed that miR-155 had considerable diagnostic power to discriminate breast cancer patients and healthy subjects, yielding an AUC of 0.801 with 65.0% sensitivity and 81.8% specificity.

In this study, we further explore the potential impacts of general breast cancer treatment, involving both tumor resection and chemotherapy, on serum miR-155. Similar to our findings of declined serum miR-155 levels in post-operative samples, previous studies have described these declined trends, such as miR-184 in squamous cell carcinoma [30], miR-17-3p and miR-92 in colorectal cancer [31], miR-18a in pancreatic cancer [12], miR-122 in hepatocellular carcinoma [32]. The decreased levels of miRNAs provide solid evidence that certain miRNA signatures have considerable correlations with tumor dynamics. Following curative resection, most patients with invasive breast cancer currently receive adjuvant therapy. Surprisingly, for the first time we observed declined expression of target miRNA in serum after chemotherapy, which reached the levels comparable with that of healthy subjects. The serial serum samples revealed oscillations in the level of miR-155 that correlated with the course of treatment. We propose that the curative resection and administration of anticancer agents lead to continuous reduction of tumor burden, the level of miR-155 in turn decreased.

In the general management of cancer, it is always important to monitor treatment efficacy. Under the circumstances that there is an absence of readily detectable lesions, increased levels of CA 15-3 or CEA may indicate treatment failure [3], [4]. However their clinical applications are confined, for spurious increase in levels of serum markers may occur within 6–12 weeks after the initiation of chemotherapy, as a result from drug-induced cell death [3]. Due to the undetermined half life of CA 15-3, these surges may interfere with the outcome estimation [33]. In concordance of previous findings, we found the majority of patients (72.4%) experienced elevated levels of CA 15-3 after chemotherapy. By contrast, 79% of all subjects that experienced stable disease or remission after chemotherapy showed decreased levels of serum miR-155. The same elevated trends were observed in CEA and TPS. It is not surprising that these commonly applied assays provided less precise results, for most participants in our study were stage II/III breast cancer patients. According to the last update of the American Society of Clinical Oncology (ASCO), CA 15-3 and CEA are recommended to monitor for patients with metastatic breast cancer [4]. It seems that miR-155 assay is more sensitive and indicative to the changes of tumor burden in this cohort of subjects, which raises the possibility to use miR-155 to monitor treatment, irrespective of the stage of disease. On the other hand, the results indicate a shorter half life of serum miR-155 compared with CA 15-3, CEA and TPS.

Moreover, it is of interest to note the subject that developed early relapsed lung metastasis after surgery. Actually, the removal of primary tumor has been demonstrated to promote metastases in some cases [34]. The macrometastatic cell proliferation may be primed either by “a growth-stimulating factor” or by wound-derived factors [34]–[37]. In this patient, corresponding to lung metastasis, we observed a sharp increase in the level of miR-155, suggesting that miR-155 may be actively participate in metastatic growth. As reported, microRNAs can be selectively secreted into the circulation via small membrane vesicles such as exosomes [38]. These exosomes that enrich circulating miRNAs conduct intercellular communications [39], [40]. Taylor et al. observed similar miRNA profiling between circulating exosomes and tumor biopasy [41]. Alternatively, Ohshima et al. demonstrated that let-7 miRNA family was released from metastatic gastric cancer cell line into culture media [42]. Based on these findings, it is very likely that miR-155 was packaged into exosomes, which subsequently acts as one of the growth-stimulating factors of tumor cells. On the other hand, the metastatic growth may in turn promote secretion of miR-155 from metastatic site. Corresponding to the successful control of metastatic site, we observed that serum miR-155 returned to the preoperative level.

Obviously, the ongoing studies on circulating miRNA profiles offer an exciting envision. For a reliable miRNA biomarker in circulation will dramatically facilitate the management of breast cancer. However, the development of miRNAs biomarker is cumbersome because of the current lack of assurance for accurate measurement. A major concern is the different normalization strategies. MiR-16 may not be an ideal internal control for it is not always consistent across cases and controls [13], [43], and particularly susceptible to hemolysis [17]. In our study, we took advantage of spiked-in cel-miR-39 for normalization. It is not without limitation; for these synthetic miRNAs are less stable than endogenous miRNAs [44]. Recently, Hu Z et al. [45] applied Solexa sequencing and TaqMan Low Density Array and found that the combination of miR-484 and miR-191 was optimal to work as endogenous control for most diagnosed tumors. However, whether these endogenous miRNAs are still suitable for normalization after general management warrants further evaluation. In addition, although the concentrations of circulating miRNA are independent of age and gender, different racial expression profiles have been recently reported [46], [47], which raises the question of whether differential genetic background and environmental variations such as nutrition and exercise would affect the abundance of miRNAs in the circulation.

Taken together, this study extends the findings of previous studies about the serum levels of miR-155 in breast cancer patients. Our data provide complementary information on its diagnostic value. From another respect, new insights into the oscillations in the level of miR-155 following the course of therapy are setting the stage for further diagnostic innovations, with the goal of monitoring treatment or assessing tumor dynamics. However, whether this correlation is exactly proportional requires carefully scrutiny.

## Supporting Information

Figure S1 **Serum levels of CA15-3, CEA, and TPS in healthy controls (HC) compared with that of in breast cancer (BC).** Box plots represent the levels of CA15-3, CEA, and TPS using the log data to accommodate the wide range. Statistically significant differences were determined using Mann-Whitney tests. There was no significant difference in serum concentrations of CA 15-3 (*p* = 0.9607), CEA (*p* = 0.0528) and TPS (*P* = 0.4046) in cases versus controls. The median values in the control group were 9.78 U/L (range; 1.80–24.24), 1.73 ng/mL (range; 0.50–4.33), and 61.09 U/mL (range; 20.60–662.96), respectively; the median values in the case group were 9.86 U/L (range; 1.76–48.00), 1.46 ng/mL (range; 0.20–20.46), and 60.25 U/mL (range; 8.97–546.00).
(TIF)

Table S1 **Characteristics of 29 patients for serial measurements after curative resection and chemotherapy.**
(DOC)

## References

[1] Anonymous (1996) Clinical practice guidelines for the use of tumor markers in breast and colorectal cancer. Adopted on May 17, 1996 by the American Society of Clinical Oncology. J Clin Oncol 14: 2843–2877. 8874347 10.1200/JCO.1996.14.10.2843

[2] Guadagni F, Ferroni P, Carlini S, Mariotti S, Spila A, et al (2001) A re-evaluation of carcinoembryonic antigen (CEA) as a serum marker for breast cancer: a prospective longitudinal study. Clin Cancer Res 7: 2357–2362. 11489813

[3] Duffy MJ, Evoy D, McDermott EW (2010) CA 15–3: uses and limitation as a biomarker for breast cancer. Clin Chim Acta 411: 1869–1874. 20816948 10.1016/j.cca.2010.08.039

[4] Harris L, Fritsche H, Menel R, Norton L, Ravdin P, et al (2007) American Society of Clinical Oncology 2007 Update of recommendations for the use of tumor markers in breast cancer. J Clin Oncol 25: 5287–5312. 17954709 10.1200/JCO.2007.14.2364

[5] Sturgeon CM, Duffy MJ, Stenman UK, Lilja H, Brünner N, et al (2008) National Academy of Clinical Biochemistry Laboratory Medicine practice guidelines for use of tumor markers in testicular, prostate, colorectal, breast and ovarian cancers. Clin Chem 54: e11–79. 19042984 10.1373/clinchem.2008.105601

[6] D’Alessandro R, Roselli M, Ferroni P, Mariotti S, Spila A, et al (2001) Serum tissue polypeptide specific antigen (TPS): a complementary tumor marker to CA 15–3 in the management of breast cancer. Breast Cancer Res Treat 68: 9–19. 11678313 10.1023/a:1017903724176

[7] Cortez MA, Bueso-Ramos C, Ferdin J, Lopez-Berestein G, Sood AK, et al (2011) MicroRNAs in body fluids–the mix of hormones and biomarkers. Nat Rev Clin Oncol 8: 467–477. 21647195 10.1038/nrclinonc.2011.76 PMC3423224

[8] Schwarzenbach H, Hoon DS, Pantel K (2011) Cell-free nucleic acids as biomarkers in cancer patients. Nat Rev Cancer 11: 426–437. 21562580 10.1038/nrc3066

[9] Kosaka N, Iguchi H, Ochiya T (2010) Circulating microRNA in body fluid: a new potential biomarker for cancer diagnosis and prognosis. Cancer Sci 101: 2087–2092. 20624164 10.1111/j.1349-7006.2010.01650.x PMC11159200

[10] Brase JC, Wuttig D, Kuner R, Sültmann H (2010) Serum microRNAs as non-invasive biomarkers for cancer. Mol Cancer 9: 306. 21110877 10.1186/1476-4598-9-306 PMC3002336

[11] Mitchell PS, Parkin RK, Kroh EM, Fritz BR, Wyman SK, et al (2008) Circulating microRNAs as stable blood-based markers for cancer detection. Proc Natl Acad Sci U S A 105: 10513–10518. 18663219 10.1073/pnas.0804549105 PMC2492472

[12] Morimura R, Komatsu S, Ichikawa D, Takeshita H, Tsujiura M, et al (2011) Novel diagnostic value of circulating miR-18a in plasma of patients with pancreatic cancer. Br J Cancer 105: 1733–1740. 22045190 10.1038/bjc.2011.453 PMC3242609

[13] Tomimaru Y, Equchi H, Nagano H, Wada H, Kobayashi S, et al (2012) Circulating microRNA-21 as a novel biomarker for hepatocellular carcinoma. J hepatol 56: 167–175. 21749846 10.1016/j.jhep.2011.04.026

[14] Shen J, Todd NW, Zhang H, Yu L, Lingxiao X, et al (2011) Plasma microRNAs as potential biomarkers for non-small-cell lung cancer. Lab Invest 91: 579–587. 21116241 10.1038/labinvest.2010.194 PMC3130190

[15] Wang F, Zheng Z, Guo J, Ding X (2010) Correlation and quantification of microRNA aberrant expression in tissues and sera from patients with breast tumor. Gynecol Oncol 119: 586–593. 20801493 10.1016/j.ygyno.2010.07.021

[16] Chen X, Ba Y, Ma L, Cai X, Yin Y, et al (2008) Characterization of microRNAs in serum: a novel class of biomarkers for diagnosis of cancer and other diseases. Cell Res 18: 997–1006. 18766170 10.1038/cr.2008.282

[17] McDonald JS, Milosevic D, Reddi HV, Grebe SK, Algeciras-Schimnich A (2011) Analysis of circulating microRNA: preanalytical and analytical challenges. Clin Chem 57: 833–840. 21487102 10.1373/clinchem.2010.157198

[18] Jiang S, Zhang HW, Lu MH, He XH, Li Y (2010) MicroRNA-155 functions as an OncomiR in breast cancer by targeting the suppressor of cytokine signaling 1 gene. Cancer Res 70: 3119–3127. 20354188 10.1158/0008-5472.CAN-09-4250

[19] Iorio MV, Ferracin M, Liu CG, Veronese A, Spizzo R (2005) MicroRNA gene expression deregulation in human breast cancer. Cancer Res 65: 7065–7070. 16103053 10.1158/0008-5472.CAN-05-1783

[20] Zhu W, Qin W, Atasoy U, Sauter ER (2009) Circulating microRNAs in breast cancer and healthy subjects. BMC Res Notes 2: 89. 19454029 10.1186/1756-0500-2-89 PMC2694820

[21] Roth C, Rack B, Müller V, Janni W, Pantel K (2010) Circulating microRNAs as blood-based markers for patients with primary and metastatic breast cancer. Breast Cancer Res 12: R90. 21047409 10.1186/bcr2766 PMC3046429

[22] Heneghan HM, Miller N, Kerin MJ (2011) Circulating microRNAs: promising breast cancer Biomarkers. Breast Cancer Res 13: 402. 21345257 10.1186/bcr2798 PMC3109560

[23] Heneghan HM, Miller N, Lowery AJ, Sweeney KJ, Newell J, et al (2010) Circulating microRNAs as novel minimally invasive biomarkers for breast cancer. Ann Surg 25: 499–505. 10.1097/SLA.0b013e3181cc939f 20134314

[24] Miller AB, Hoogstraten B, Staquet M, Winkler A (1981) Reporting results in cancer treatment. Cancer 47: 207–214. 7459811 10.1002/1097-0142(19810101)47:1<207::aid-cncr2820470134>3.0.co;2-6

[25] Livak KJ, Schmittgen TD (2001) Analysis of relative gene expression data using real-time quantitative PCR and the 2(-Delta Delta C(T)) Method. Methods 25: 402–408. 11846609 10.1006/meth.2001.1262

[26] Mattiske S, Suetani RJ, Neilsen PM, Callen DF (2012) The Oncogenic Role of miR-155 in Breast cancer. Cancer Epidermiol Biomarkers Prev [Epub ahead of print]. 10.1158/1055-9965.EPI-12-0173 22736789

[27] Wang J, Hua HJ (2012) Role of miR-155 in breast cancer. Front Biosci 17: 2350–2355. 10.2741/4056 22652783

[28] Volinia S, Calin GA, Liu CG, Ambs S, Cimmino A, et al (2006) A microRNA expression signature of human solid tumors defines cancer gene targets. Proc Natl Acad Sci U S A 103: 2257–2261. 16461460 10.1073/pnas.0510565103 PMC1413718

[29] Pritchard CC, Kroh E, Wood B, Arroyo JD, Dougherty KJ, et al (2012) Blood cell origin of circulating microRNAs: a cautionary note for cancer biomarker studies. Cancer Prev Res (Phila) 5: 492–497. 22158052 10.1158/1940-6207.CAPR-11-0370 PMC4186243

[30] Wong TS, Liu XB, Wong BY, Ng RW, Yuen AP, et al (2008) Mature miR-184 as Potential Oncogenic microRNA of Squamous Cell Carcinoma of Tongue. Clin Cancer Res 14: 2588–2592. 18451220 10.1158/1078-0432.CCR-07-0666

[31] Ng EK, Chong WW, Jin H, Lam EK, Shin VY, et al (2009) Differential expression of microRNAs in plasma of patients with colorectal cancer: a potential marker for colorectal cancer screening. Gut 58: 1375–1381. 19201770 10.1136/gut.2008.167817

[32] Qi P, Cheng SQ, Wang H, Li N, Chen YF, et al (2011) Serum microRNAs as biomarkers for hepatocellular carcinoma in Chinese patients with chronic hepatitis B virus infection. PLoS One 6: e28486. 22174818 10.1371/journal.pone.0028486 PMC3234251

[33] Wu SC, Chou FF, Rau KM (2010) Clinical significance of a serum CA 15–3 surge and the usefulness of CA 15–3 kinetics in monitoring chemotherapy response in patients with metastatic breast cancer. Breast Cancer Res Treat 124: 879–882. 20721690 10.1007/s10549-010-1117-3

[34] Demicheli R, Retsky MW, Hrushesky WJ, Baum M (2007) Tumor dormancy and surgery-driven interruption of dormancy in breast cancer: learning from failures. Nat Clin Pract Oncol 4: 699–710. 18037874 10.1038/ncponc0999

[35] Fisher B, Gunduz N, Coyle J, Rudock C, Saffer E (1989) Presence of a growth-stimulating factor in serum following primary tumor removal in mice. 49: 1996–2001. 2702641

[36] Van Dierendonck JH, Keijzer R, Cornelisse CJ, Van de Velde CJ (1991) Surgically induced cytokinetic responses in experimental rat mammary tumor models. Cancer 68: 759–767. 1855176 10.1002/1097-0142(19910815)68:4<759::aid-cncr2820680417>3.0.co;2-2

[37] Abramovitch R, Marikovsky M, Meir G, Neeman M (1998) Stimulation of tumour angiogenesis by proximal wounds: spatial and temporal analysis by MRI. Br J Cancer 77: 440–447. 9472641 10.1038/bjc.1998.70 PMC2151289

[38] Gallo A, Tandon M, Alevizos I, Illei GG (2012) The majority of microRNAs detectable in serum and saliva is concentrated in exosomes. PLoS One 7: e30679. 22427800 10.1371/journal.pone.0030679 PMC3302865

[39] Valadi H, Ekström K, Bossios A, Sjöstrand M, Lee JJ, et al (2007) Exosome-mediated transfer of mRNAs and microRNAs is a novel mechanism of genetic exchange between cells. Nat Cell Biol 9: 654–659. 17486113 10.1038/ncb1596

[40] Kosaka N, Iguchi H, Yoshioka Y, Takeshita F, Matsuki Y, et al (2010) Secretory mechanisms and intercellular transfer of microRNAs in living cells. J Biol Chem 285: 17442–17452. 20353945 10.1074/jbc.M110.107821 PMC2878508

[41] Taylor DD, Gercel-Taylor C (2008) MicroRNA signatures of tumor-derived exosomes as diagnostic biomarkers of ovarian cancer. Gynecol Oncol 110: 13–21. 18589210 10.1016/j.ygyno.2008.04.033

[42] Ohshima K, Inoue K, Fujiwara A, Hatakeyama K, Kanto K, et al (2010) Let-7 microRNA family is selectively secreted into the extracellular environment via exosomes in a metastatic gastric cancer cell line. PloS One 5: e13247–56. 20949044 10.1371/journal.pone.0013247 PMC2951912

[43] Lou Y, Yang X, Wang F, Cui Z, Huang Y (2010) MicroRNA-21 promotes the cell proliferation, invasion and migration abilities in ovarian epithelial carcinomas through inhibiting the expression of PTEN protein. Int J Mol Med 26: 819–827. 21042775 10.3892/ijmm_00000530

[44] Creemers EE, Tijsen AJ, Pinto YM (2012) Circulating MicroRNAs: Novel Biomarkers and Extracellular Communicatiors in Cardiovascular Disease? Circ Res 110: 483–495. 22302755 10.1161/CIRCRESAHA.111.247452

[45] Hu Z, Dong J, Wang LE, Ma H, Liu J, et al. (2012) Serum microRNA profiling and breast cancer risk: the use of miR-484/191 as endogenous controls. Carcinogenesis. In press. 10.1093/carcin/bgs030 22298638

[46] Zhao H, Shen J, Medico L, Wang D, Ambrosone CB, et al (2010) A pilot study of circulating miRNAs as potential biomarkers of early stage breast cancer. PLoS One 5: e13735. 21060830 10.1371/journal.pone.0013735 PMC2966402

[47] Heegaard NH, Schetter AJ, Welsh JA, Yoneda M, Bowman ED, et al (2012) Circulating micro-RNA expression profiles in early stage nonsmall cell lung cancer. Int J Cancer 130: 1378–1386. 21544802 10.1002/ijc.26153 PMC3259258

