# Supplementary material for: Serum MicroRNA-155 as a Potential Biomarker to Track Disease in Breast Cancer
Source: PLoS One. 2012 Oct 10;7(10):e47003. doi: 10.1371/journal.pone.0047003 (PMC3468565; doi:10.1371/journal.pone.0047003)
Supplement: Table S1 — Characteristics of 29 patients for serial measurements after curative resection and chemotherapy. (DOC) [file pone.0047003.s002.doc]

Table S1. Characteristics of 29 patients for serial measurements after curative resection and chemotherapy

| **Characteristics** | **Breast cancer (n = 29)** |
| --- | --- |
| **Age** |  |
| Mean | 50 |
| Median (range) | 53 (26-75) |
| **TNM stage** |  |
| Ⅰ | 3 |
| Ⅱ | 15 |
| Ⅲ | 10 |
| Ⅳ | 1 |
| **T classification** |  |
| T_1_ | 12 |
| T_2_ | 17 |
| **Nodal status** |  |
| Negative | 9 |
| Positive | 20 |
| **ER** |  |
| Negative | 7 |
| Positive | 21 |
| Undetermined | 1 |
| **PR** |  |
| Negative | 6 |
| Positive | 20 |
| Undetermined | 3 |
| **HER2** |  |
| Negative | 24 |
| Positive | 5 |
| **Subtype** |  |
| Luminal | 22 |
| HER2 | 1 |
| Triple negative | 3 |
| Undetermined | 3 |
